# Thermal properties of ruthenium alkylidene-polymerized dicyclopentadiene

**DOI:** 10.3762/bjoc.11.159

**Published:** 2015-08-21

**Authors:** Yuval Vidavsky, Yotam Navon, Yakov Ginzburg, Moshe Gottlieb, N Gabriel Lemcoff

**Affiliations:** 1 Department of Chemistry, Ben-Gurion University of the Negev Beer Sheva-84105, Israel; 2 Department of Chemical Engineering, Ben-Gurion University of the Negev Beer Sheva-84105, Israel

**Keywords:** glass-transition temperature, polydicyclopentadiene, ring opening metathesis polymerization, ruthenium-catalyzed olefin metathesis, thermoset polymers

## Abstract

Differential scanning calorimetry (DSC) analysis of ring opening methatesis polymerization (ROMP) derived polydicyclopentadiene (PDCPD) revealed an unexpected thermal behavior. A recurring exothermic signal can be observed in the DSC analysis after an elapsed time period. This exothermic signal was found to be proportional to the resting period and was accompanied by a constant increase in the glass-transition temperature. We hypothesize that a relaxation mechanism within the cross-linked scaffold, together with a long-lived stable ruthenium alkylidene species are responsible for the observed phenomenon.

## Introduction

**Figure 1:**
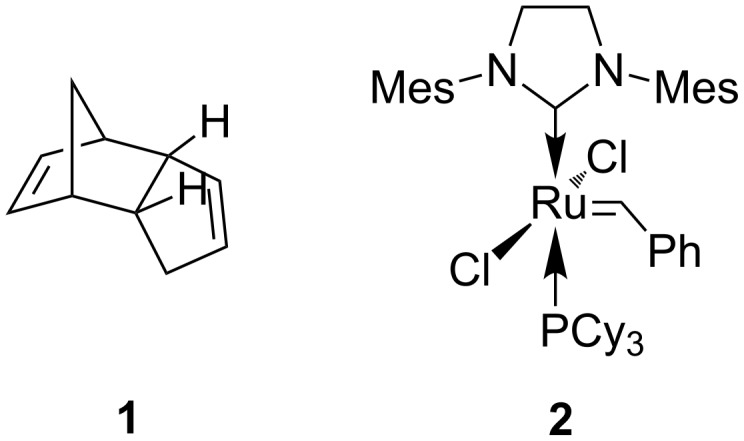
DCPD (**1**) and ruthenium benzylidene catalyst **2**.

Olefin metathesis [1–6] has advanced to become a major synthetic tool in academia [7–11] and industry [12]. Metathesis polymerization techniques [13–15], and especially ring opening metathesis polymerization (ROMP) [16–17], have had a vital role in this growth. Polydicyclopentadiene (PDCPD), probably the most widely used metathesis polymer, is formed through ROMP of mostly endo-dicyclopentadiene (DCPD, **1**) (Figure 1). The Grubbs-type ruthenium initiators, known for their high activity, stability and functional group tolerance are extensively used to promote this type of olefin metathesis reactions. For example, the Grubbs second generation catalyst **2** [18] (Figure 1), may be used to initiate ROMP reactions of suitable strained cycloolefins.

**Scheme 1:**
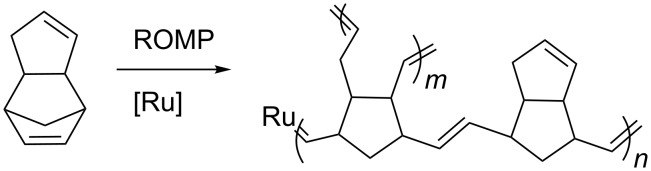
ROMP of dicyclopentadiene by a ruthenium alkylidene initiator.

DCPD is a common byproduct in the naphtha cracking process [19] and has two carbon–carbon double bonds, which readily undergo ROMP reactions with ruthenium alkylidenes. By adding the appropriate initiator, the highly strained and reactive norbornene double bond can be disrupted first to afford a linear polymer, followed by the ring opening of the less reactive cyclopentene double bond to effectively cross-link the chains (Scheme 1). Notably, with tungsten and molybdenum initiators the linear polymer may be isolated [20–21]; unlike the case with ruthenium initiators where only cross-linked polymers are obtained. This polyolefinic cross-linked thermoset material exhibits outstanding thermal stability [22], mechanical strength [23], fracture toughness [24] and dielectric characteristics [25]. Thanks to these properties PDCPD has become a very attractive polymer for several applications and is one of the most ubiquitous ROMP materials in industrial uses.

The relatively new PDCPD polymer has been widely explored for its thermal properties over the past decade. Cao et al. [26] reported glass-transition temperatures (*T*_g_) as high as 165 °C and total conversions of 98.9% at polymerization temperatures of 60 °C with the Grubbs first generation catalyst. By carrying out detailed differential scanning calorimetry (DSC) analyses Kessler and Mauldin [27] demonstrated a sharp exothermic peak related to the heat of reaction and a final *T*_g_ of 164 °C at conversions of 90% right after curing. Dimonie et al. [28–29] examined the nature of the first exothermic peak of linear PDCPD using DSC and showed thermal polymerization completion after 2 h at 150 °C as the exothermic peak disappeared given these conditions. Kessler and White [30] also explored the cure kinetics of the polymer using DSC and reached a *T*_g_ of 139 °C for a "fully cured" product. In addition, Lee et al. [31] showed the absence of the exothermic peak on a second DSC scan, revealing a *T*_g_ as high as 160 °C. While literature glass-transition temperatures range from 140 to 165 °C, the polymer's thermal behavior for extended periods of time is not usually reported. Understanding this behavior is crucial for a polymer with a wide range of engineering applications in order to ensure the effectiveness and long-standing stability of the polymer. In this work we examined the thermal behavior over time of PDCPD obtained by ruthenium-induced ROMP of DCPD.

## Results and Discussion

The observation of recurrent exothermic peaks in calorimetric analyses and a continuous rise in glass-transition temperature over time led us to study this phenomenon and propose a plausible mechanism for this behavior.

**Figure 2:**
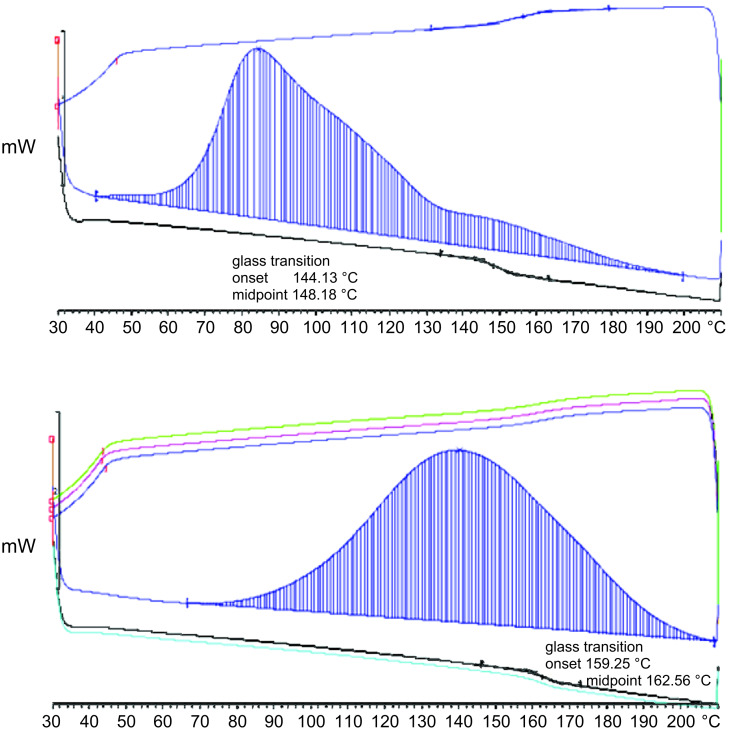
Top: DSC plot of PDCPD 24 hours after polymerization. Blue line: 1st heating–cooling cycle. Black line: 2nd cycle. Bottom: DSC of PDCPD sample after two weeks. Blue line shows the reappearing exothermic peak (1st cycle). The black and cyan lines show the elevation in *T*_g_ and disappearance of the exothermic peak.

When a sample of PDCPD produced by ROMP of DCPD with catalyst **2** was initially subjected to a differential scanning calorimeter (DSC) run cycle, a strong exothermic peak was observed, which was at first associated with the reaction of remaining DCPD according to previous studies. Fourteen further DSC cycles were run immediately and, as expected, no exothermic peak was observed (Figure 2, top). The glass-transition temperature (*T*_g_) signature was observed at 148 °C, with good correlation to literature values (vide supra).

As stated before, we desired to monitor the *T*_g_ over time; thus, the same sample was subjected to an additional identical DSC cycle two weeks later. To our surprise, an exothermic peak reappeared and the *T*_g_ value was recorded at 162.6 °C (Figure 2, bottom). Carrying out the measurement and the subsequent storage under nitrogen atmosphere afforded the same results. DSC analyses were then repeated with a number of polymer samples and the ‘return’ of the exothermic peak after prolonged time periods was found to be completely reproducible, a finding which led us to further investigate this phenomenon.

**Figure 3:**
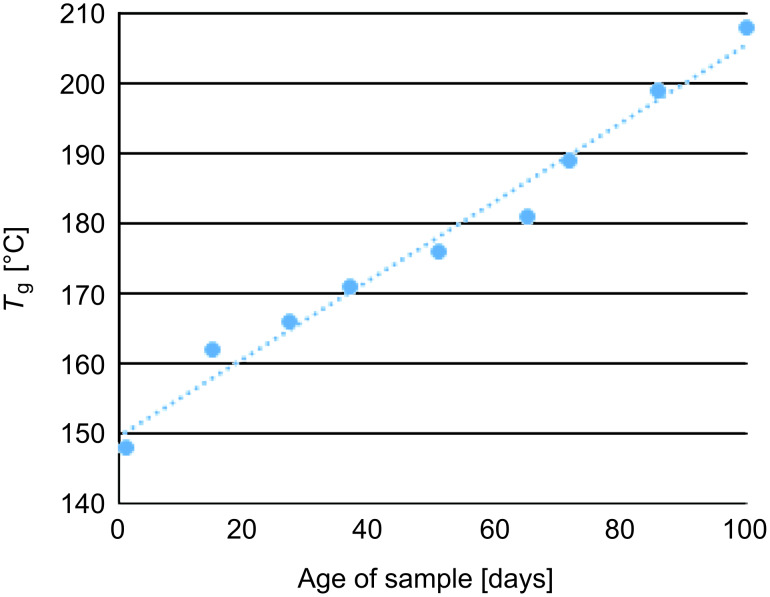
Change in *T*_g_ for a representative PDCPD sample as a function of time.

Thus, a set of PDCPD samples was subjected to a series of DSC heating–cooling cycles, with resting periods at room temperature between the cycles. During a period of 120 days the *T*_g_ constantly increased with every rest period until its value could not be further detected by the DSC analysis. A maximum glass-transition temperature was recorded at approximately 210 °C, which is to our knowledge the highest *T*_g_ recorded for PDCPD in the scientific literature (Figure 3).

**Figure 4:**
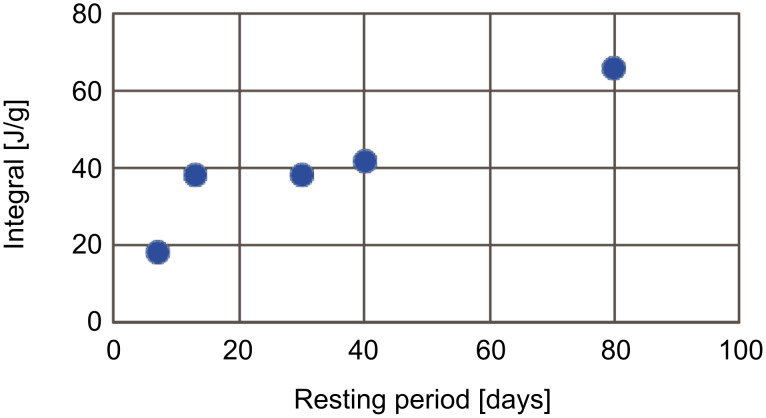
Intensity of exothermic peak as a function of rest time at room temperature for different samples.

The rise of the *T*_g_ after the rest periods was permanently accompanied by the reappearance of the exothermic peak. It was furthermore observed that the sample with the longest rest period of 16 months at room temperature showed the largest exothermic peak. The intensity of the exothermic peak was strongly correlated to the rest time between the analyses, where longer resting periods gave larger exothermic peaks (Figure 4) and very short time periods (such as the immediate repetition) did not afford any exothermic signal at all. For instance, a sample that was rested for 16 months without heating showed an extremely strong exothermic peak with a value of 151 J/g, even larger than the peaks observed at the first measurement.

**Figure 5:**
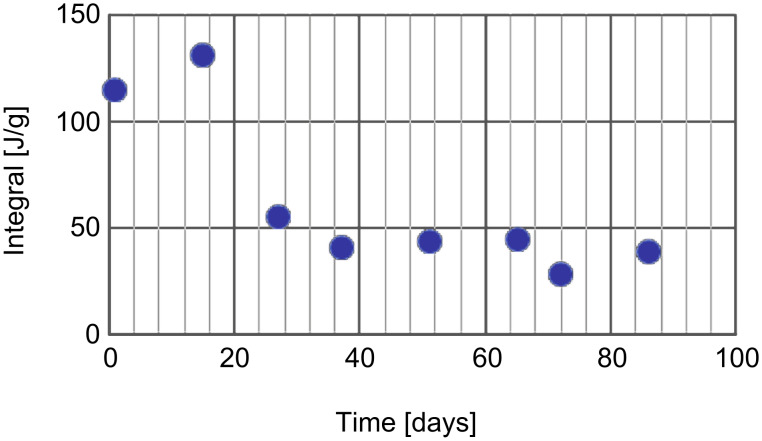
Peak intensity as function of age. Samples were analyzed every two weeks. The abnormal low intensity of the peak after 72 days is due to one week rest time instead of the regular two weeks rest time. The high intensity observed in the first two measurements may be attributable to further polymerization of unreacted cyclopentene bonds and free monomer.

A sample that was allowed equal rest periods of two weeks (Figure 5) showed very similar exothermic integrals (ca. 40 J/g), even after 90 days, except for the first two abnormally high peaks (probably due to reaction of unreacted strained cycloalkenes in the sample).

It is important to note that the samples were always weighed between heating cycles and the weight of the crucible and polymer remained unchanged throughout the experiment. As the effect of resting time was established, we proceeded to study whether the resting temperature would influence the observed exothermic signal and the resultant *T*_g_. Therefore, a set of samples was prepared, similar DSC cycles were run but this time, the samples were rested at different temperatures, i.e., room temperature, −5 °C, and at −196 °C (liquid nitrogen).

**Figure 6:**
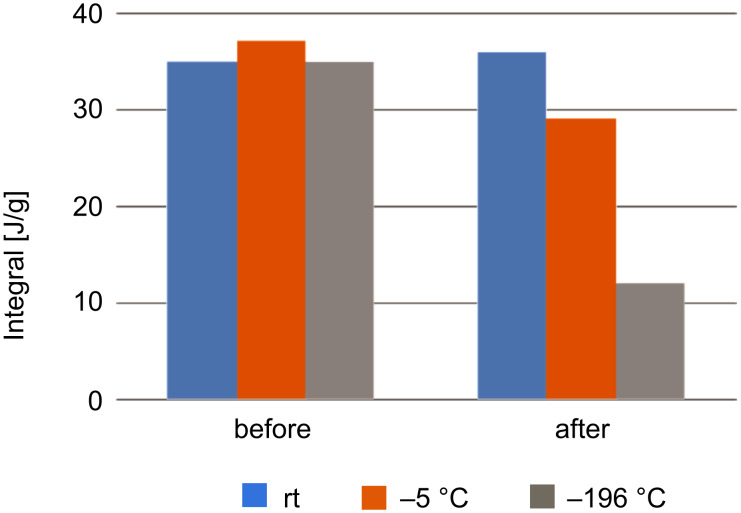
Resting temperature effect. Blue columns: resting at room temperature. Orange columns: resting at −5 °C. Gray columns: resting at −196 °C. Time elapsed between measurements was 1 week.

As shown in Figure 6, only extreme cooling using liquid nitrogen reduced the peak intensity significantly by 63%, compared to ambient temperature. Storage of the sample at −5 °C still afforded a relatively strong exothermic signal.

According to the ROMP mechanism, a ruthenium alkylidene species may remain entrapped within the PDCPD matrix. The data collected led us to assume that the exothermic peak may arise from an internal metathetic process which occurs only after the polymer microstructure equilibrates and further ruthenium–alkylidene metathesis with neighboring double bonds may be promoted. Alternatively, thermal decomposition of DCPD (or larger oligomers) to cyclopentadiene (CPD) by a retro-Diels–Alder reaction could also explain the observed phenomenon, although unlikely at room temperature. Both hypotheses were tested.

CPD is less reactive in metathesis reactions than DCPD, and will gradually dimerize at ambient conditions to give the latter. In order to estimate a possible formation of CPD as the reason for the observed thermal behavior, a series of DCPD samples with different percentages of externally added CPD were subjected to heat–cool–rest cycles. Table 1 shows the lack of correlation between the amount of CPD in the sample and the glass-transition temperature increase. Moreover, the presence of volatile monomers such as DCPD and, even more so CPD, after heating cycles and long periods of time is highly improbable.

**Table 1:** *T*_g_ dependence on CPD content (%).

Entry^a^	vol % CPD	1st *T*_g_^b^	2nd *T*_g_^c^

1	0	148	162
2	2.5	142	158
3	10	158	165

^a^Conditions: 0.5 mg of **2** in 0.1 mL CH_2_Cl_2_; 10 mL of monomer/s. ^b^First DSC run after 24 hours at 60 °C. ^c^Second DSC run after two weeks at room temperature.

**Figure 7:**
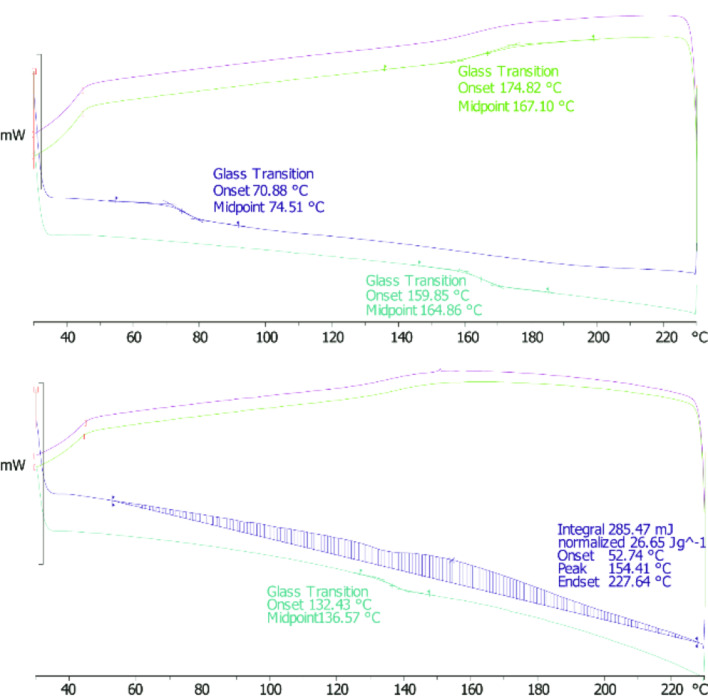
Top: Sample after 1 week with ethyl vinyl ether. Bottom: Sample after 1 week with diethyl ether.

As mentioned before, we hypothesized that the exothermic signal reemerged due to secondary metathesis reactions which can occur after polymer relaxation and repositioning of the active ruthenium alkylidene within the cross-linked polymer network. To validate this assumption a sample in the DSC crucible was flooded with ethyl vinyl ether for 5 days, trying to deactivate any remaining catalytic species by formation of inert Fischer carbene [32]. A control sample was flooded with diethyl ether. To our satisfaction, in the sample treated with ethyl vinyl ether the exothermic peak was suppressed while the control experiment (with diethyl ether) behaved as indicated in previous experiments (Figure 7). These results support the theory that an olefin metathesis reaction is occurring and that it is the source of the observed exothermic peak.

## Conclusion

In summary, the ruthenium-catalyzed DCPD polymerization produced a polymer with an unexpected thermal behavior over long periods of time. The DSC analysis after polymerization showed a large exothermic peak, which was initially assigned to exothermicity of ROMP of unreacted cycloalkane. However, this peak reappeared repeatedly after defined resting periods (days–weeks). Our study suggests that a relaxation process is occurring within the polymer and that a long-lived catalytic species inside the polymer may still be active after prolonged periods of time. Additionally, we showed that by repeating the heating–cooling cycles over time an unprecedented glass-transition temperature for PDCPD of 210 °C was obtained. This is to the best of our knowledge the highest *T*_g_ for PDCPD recorded so far. Ongoing efforts in the lab are geared towards further elucidating the mechanism and possible applications of these observations.

## Experimental

All commercially available chemicals were of reagent grade quality and used without further purification, unless described. Differential scanning calorimetry (DSC) data was obtained using a METTLER-TOLEDO DSC 823 and results were evaluated with the STARe software. All experiments were performed with a nitrogen flow of 80 mL/min at a heating rate of 5 °C/min. Each sample was subjected to 2–3 heating–cooling cycles.

### Polymerization procedures

*endo*-Dicyclopentadiene (10 mL, 74 mmol) and a stirring magnet were added to a 20 mL vial and kept at 40 °C in order to melt the monomer. In a separate 2 mL vial, initiator **2** (0.5 mg, 5.9 × 10^−4^ mmol) was dissolved in dichloromethane (100 μL). The dissolved initiator was then transferred by syringe to the vial containing the monomer upon vigorous stirring and a 10–15 μL sample was immediately placed in a DSC 40 μL aluminum crucible. Because the monomer mixture solidifies as it comes into contact with the crucible, the latter was warmed up to 40 °C to ensure a uniform coverage on the surface of the crucible. The crucible was then sealed with an aluminum cap, and stored at 60 °C for 24 hours for complete curing of the PDCPD. During resting periods the crucible was stored at room temperature under ambient conditions unless otherwise noted.
